# Determinants of Bronchiectasis Among Patients Attending Chest Clinic of Teaching Hospital in Ethiopia: Case‐Control Study

**DOI:** 10.1155/pm/5549973

**Published:** 2026-05-11

**Authors:** Belay shewaye Mola, Mengesha Akale Tekle, Wubshet Abraham Alemu

**Affiliations:** ^1^ Department of Internal Medicine, St. Paul′s Hospital Millennium Medical college, Addis Ababa, Ethiopia; ^2^ Department of Internal Medicine, Arsi university, College of Health Sciences, Asella, Ethiopia, arsiun.edu.et

**Keywords:** bronchiectasis, case-control, determinants, Saint Paul′s Hospital Millennium Medical College

## Abstract

**Background:**

Bronchiectasis is a chronic lung disease characterized by cough and purulent sputum, recurrent infections, and airway damage. It affects people of all ages and is associated with considerable morbidity and mortality.

**Objectives and Methods:**

A case‐control study was conducted to assess factors associated with bronchiectasis. Each patient with HRCT‐confirmed bronchiectasis who visited the chest clinic was selected as a case, whereas age‐ and sex‐matched patients without bronchiectasis were selected as controls. Data was collected using a chart review and questionnaire from April 1, 2022, to June 30, 2022. Then, data was entered and analyzed using SPSS Version 25. Frequencies and cross‐tabulations were used to summarize descriptive statistics of the data. Chi‐square tests and logistic regression were done to establish associations for variables.

**Results:**

The study included 77 cases and 153 controls, with 49.35% of the cases and 33.99% of the controls being between the ages of 41 and 60. After adjusting for potential confounders, a multivariable logistic regression analysis showed that four variables were independent predictors of bronchiectasis. Bronchiectasis was six times more likely to occur in patients with a history of pulmonary tuberculosis (AOR = 6.182; 95% CI (3.16–12.10), *p* < 0.001). COPD (AOR = 2.896; 95% CI = 1.460–5.746, *p* = 0.002), bronchial asthma (AOR = 2.124; 95% CI = 1.086–4.154, *p* = 0.028), and COVID‐19 (AOR = 2.786; 95% CI = 1.454–5.340, *p* = 0.002) also increased the risk of bronchiectasis by more than twofold. There was no significant association between bronchiectasis and age, sex, or smoking history.

**Conclusion and Recommendations:**

Pulmonary tuberculosis, COVID‐19, chronic obstructive pulmonary disease, and bronchial asthma are associated with the development of bronchiectasis. Attention should be given to early identification of bronchiectasis among patients with these lung diseases.

## 1. Introduction

### 1.1. Background

Bronchiectasis is a chronic lung condition marked by cough, infection, and inflammation [1]. Radiologically, it is defined by the presence of irreversible bronchial wall dilatation [2]. Population‐based studies showed the prevalence of bronchiectasis was 33 per 100,000, 139 cases per 100,000 and 67 per 100,000 people in the United Kingdom, United States, and Germany, respectively, in 2013 [3–5].

The prevalence of bronchiectasis was found to be 5% in a hospital‐based cross‐sectional study in patients > 15 years of age with chest CT‐confirmed bronchiectasis followed at Tikur Anbessa Specialized Hospital (TASH) chest unit between September 2018 and August 2019 [6]. Patients with bronchiectasis suffer from recurrent respiratory infections, exacerbations of respiratory symptoms, and repeated hospital admissions [7].

Chronic obstructive pulmonary disease, asthma, untreated chronic infection, and recurring respiratory infections can all cause bronchiectasis [6, 8–10]. The development of bronchiectasis in these groups of patients may be influenced by age, sex, and environmental factors such as smoking [4, 5, 11].

Bronchiectasis is a significant health and economic burden to the patient, family, and the health care system. It is still a significant cause of morbidity and mortality in resource‐limited settings [8, 12, 13]. Bronchiectasis is associated with decreased physical activity [12] and increased morbidity and mortality [14–17].

### 1.2. Factors Associated With Bronchiectasis

About 60% of bronchiectasis diagnoses are made in adults over the age of 70 (4). In the population‐based study in Germany, 58% of patients with bronchiectasis had the concomitant diagnosis of COPD. Females had a higher prevalence rate than males [18].

A significant number of patients with COPD (19.3%) and asthma (17.2%) were found to develop bronchiectasis, followed by tuberculosis and nontuberculosis mycobacterial infections in South Korea [19].

According to the study done in Taiwan, idiopathic bronchiectasis was the most common cause (32%), followed by postpneumonia of noncystic bronchiectasis (24%). Posttuberculosis (12%), chronic obstructive pulmonary disease (14%), and asthma (10%) were among the other causes (2%) [20].

Multiple studies conducted in different countries, such as China, Malawi, and Egypt, found that chest infections, COPD, pulmonary tuberculosis, and bronchial asthma were associated with bronchiectasis [21, 22]. Another study from TASH in Ethiopia revealed pulmonary tuberculosis as the most common etiology, followed by recurrent childhood infections [6, 23].

COVID‐19 has been shown to be an emerging cause of bronchiectasis, and the onset of bronchiectasis associated with COVID‐19 tends to be more rapid [24]. Two studies conducted in Wuhan City, China, confirmed the association of bronchiectasis and COVID survival patients [25, 26], and its prevalence was higher in patients with symptoms lasting more than 2 weeks in comparison to those followed for less than 2 weeks [27, 28].

Given the high burden of bronchiectasis and the scarcity of research on its risk factors within the country, this study is aimed at identifying the factors associated with bronchiectasis among patients attending chest clinics.

## 2. Materials and Methods

A case‐control study was done at St. Paul′s Hospital Millennium Medical College, Ethiopia′s second‐largest governmental tertiary care hospital, which is located in Addis Ababa. It serves a population of more than 5 million people as a medical facility. Daily, the hospital serves approximately 1200 patients [29].

Sample size was calculated using the formula for unmatched case‐control study, taking a 95% confidence interval, 80% power, a control‐to‐case ratio of 2, and a prevalence of bronchiectasis among patients with previous chest infections of 76.4% from Tikur Anbessa Hospital, and assuming that a 20% difference between cases and controls is clinically significant. Considering the 10% nonresponse rate, the final sample size was 230.

All adult patients attending the chest follow‐up clinic and who have documented chest HRCT findings were included. Patients with radiologic (HRCT) evidence of bronchiectasis were selected as cases, whereas patients with no evidence of bronchiectasis on HRCT were considered as controls.

During the study period, from April 1 to June 30, 2022, each patient with bronchiectasis who visited the chest clinic was selected as a case, and age‐ and sex‐matched patients without bronchiectasis were selected as controls until the required sample size was reached. Chart review was done, and for missing data, a questionnaire was used to collect further data. The checklist was pretested to learn about the appropriateness of the questions.

### 2.1. Study Variables and Data Analysis

This study considered bronchiectasis as a dependent variable, whereas tuberculosis, Covid‐19, COPD, asthma, smoking, alcohol, age, and sex were used as independent variables. The data were entered, coded, cleaned, and analyzed using the SPSS window program Version 25. Frequencies and cross tabulations were used to summarize descriptive statistics of the data, and tables and figures were used for data presentation. The chi‐square test and logistic regression were used to determine the associations between dependent and independent variables, and variables having a *p* value of less than or equal to 0.05 were considered as having an association with the dependent variable.

### 2.2. Operational Definition

Bronchiectasis is defined by a combination of clinical features and radiologic abnormalities. Clinical features indicative of bronchiectasis include cough on most days of the week, sputum production on most days of the week, and a history of exacerbations [30].

Radiologic criteria for the diagnosis of bronchiectasis include [31]:

1. Airway‐to‐arterial ratio ≥1.5 (internal airway lumen diameter/adjacent pulmonary artery diameter).
2. Lack of tapering of bronchi (tram track appearance).
3. Airway visibility within 1 cm of a costal pleural surface or touching the mediastinal pleura.

Smoker is defined as any person who has smoked at least 100 cigarettes in his/her lifetime [32].

## 3. Results

### 3.1. Sociodemographic Profile of the Studied Population

A total of 230 patients were evaluated. Among these, 77 have been diagnosed with bronchiectasis and 153 have no bronchiectasis. Sociodemographic variables were comparable between the cases and control group. The mean age of patients with bronchiectasis and without bronchiectasis was 51 (Table 1).

**Table 1:** Lists of variables among cases and control group at St. Paul′s Hospital Millennium Medical College, Addis Ababa, Ethiopia.

Variables	Bronchiectasis	
	Yes (*n* = 77)	No (*n* = 153)
Sex		
Male	38 (49.35%)	79 (51.6%)
Female	39 (50.65%)	74 (48.4%)
Age range		
< 20	2 (2.6%)	1 (0.7%)
21–40	17 (22.1%)	51 (33.33%)
41–60	38 (49.35%)	52 (33.99%)
> 60	20 (25.97%)	49 (32.02%)
Smoking history		
Yes	15 (19.48%)	18 (11.76%)
No	62 (80.52%)	135 (88.23%)
Alcoholic history		
Yes	30 (38.96%)	49 (32.02%)
No	47 (61.04%)	104 (67.98%)
Tuberculosis		
Yes	53 (68.83%)	52 (33.99%)
No	24 (31.17%)	101 (66.01%)
COPD		
Yes	40 (51.95%)	41 (26.8%)
No	37 (48.05%)	112 (73.2%)
Asthma		
Yes	45 (58.44%)	59 (38.56%)
No	32 (41.56%)	94 (61.44%)
COVID 19		
Yes	39 (50.65%)	49 (32.02%)
No	38 (49.35%)	104 (67.98%)

*Note:* Cases‐median age—53 years, mean age 51years (age range 19–80years). Controls‐median age—52 years, mean age 51 years (age range 18–86 years).

### 3.2. Frequency of Independent Variables Among Cases and Controls

**Figure 1:**
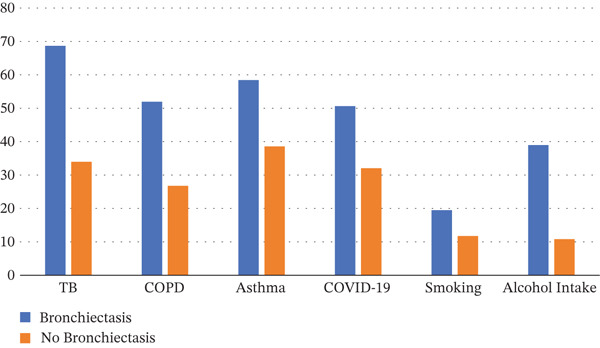
Frequency of independent variables among cases and controls.

Overall, patients with bronchiectasis had a higher frequency of tuberculosis, COPD, asthma, COVID‐19, and smoking history than patients without bronchiectasis. Figure 1 shows the detailed distribution of independent variables among cases and controls.

### 3.3. Presenting Symptoms

Patients with bronchiectasis were assessed for the presence of symptoms within 2 weeks prior to data collection. Accordingly, the majority of patients had cough (94.8%) with blood‐tinged sputum (63.6%), shortness of breath (84.4%), and easy fatigability (79.2%). Most of the patients reported significant limitation of their daily activities affecting their quality of life.

### 3.4. HRCT Findings

**Figure 2:**
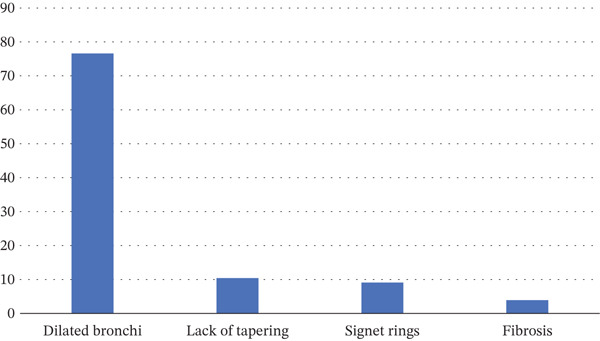
Radiologic (HRCT) Findings in patients with bronchiectasis.

The most common reported findings on CT scans of patients with bronchiectasis were dilated bronchi (76.62%) and signet rings (9.10%), followed by lack of tapering (10.39%) and fibrosis (3.90%) Figure 2.

### 3.5. Determinants of Bronchiectasis

Based on the *p* value computed, pulmonary tuberculosis (< 0.001), COPD (< 0.001), bronchial asthma (0.004), and COVID‐19 (0.006) have shown significant association with bronchiectasis, whereas there is no significant association found between smoking (0.115), drinking alcohol (0.296), and female sex (0.744).

Logistic regression was performed to determine how pulmonary tuberculosis, COPD, bronchial asthma, and COVID‐19 affect a patient′s probability of having bronchiectasis. Accordingly, both binary and multivariate regression revealed that there was a strong association with a history of tuberculosis, COVID‐19, COPD, and bronchial asthma, as depicted in Table 2. Patients with a history of TB treatment were six times more likely to develop bronchiectasis (AOR = 6.182; 95% CI = 3.163–12.083, *p* < 0.001). COPD (AOR = 2.896; 95% CI = 1.460–5.746, *p* = 0.002), bronchial asthma (AOR = 2.124; 95% CI = 1.086–4.154, *p* = 0.028), and COVID‐19 (AOR = 2.786; 95% CI = 1.454–5.340, *p* = 0.002) also increased the risk of bronchiectasis by more than twofold.

**Table 2:** Determinants of bronchiectasis among patients visiting the chest clinic at St. Paul′s Hospital Millennium Medical College, Addis Ababa, Ethiopia.

Characteristics	Case	Control	COR (95%)	AOR (95%)	*p* value
Sex					
Male	38	79	0.91 (0.63–1.243)	0.97 (0.73–1.372)	0.06
Female	39	74	Reference		
Smoking					
Yes	15	18	1.814 (1.04–4.762)	1.932 (1.240–5.031)	0.052
No	62	135	Reference		
Age					
< 40	19	52	0.895 (0.573–1.072)	0.634 (0.430–1.007)	0.051
41–60	38	52	Reference		
> 40	20	49			
Tuberculosis					
Yes	53	52	4.289 (2.385–7.714)	6.182 (3.163–12.083)	< 0.001
No	24	101	Reference		
COPD					
Yes	40	41	2.953 (1.666–5.236)	2.896 (1.460–5.746)	0.002
NO	37	112	Reference		
Asthma					
Yes	45	59	2.240 (1.282–3.915)	2.124 (1.086–4.154)	0.028
No	32	94	Reference		
COVID					
Yes	39	49	2.178 (1.243–3.818)	2.786 (1.454–5.340)	0.002
No	38	104	Reference		

Abbreviations: AOR, adjusted odds ratio; CI, confidence interval; COR, crude odds ratio.

## 4. Discussion

Ethiopia, a developing country with a high burden of tuberculosis (estimated prevalence of 12.81%) [33], faces a significant public health challenge from post‐TB sequelae such as fibrosis, bronchiectasis, cor pulmonale, and fungal balls—adding to the long‐term human suffering caused by these complications. This case‐control study was conducted to assess factors associated with bronchiectasis among 77 patients who have bronchiectasis and 153 who have no bronchiectasis. Accordingly, a significant association was found between bronchiectasis and pulmonary tuberculosis, COPD, bronchial asthma, and COVID‐19. In contrast to other studies, age, sex, and smoking history showed no association with bronchiectasis. The majority of patients who develop bronchiectasis in developing countries have postinfection causes, mainly from pulmonary tuberculosis. This is in contrast to data from the developed world, where asthma and COPD predominate as risk factors for noncystic bronchiectasis.

Pulmonary tuberculosis may cause bronchiectasis by multiple mechanisms. The primary mechanism is tuberculous bronchitis [34]. Other mechanisms include endobronchial involvement leading to localized bronchial obstruction and fibrosis, extrinsic bronchial compression by tuberculous lymphadenopathy, and decreased pulmonary compliance caused by parenchymal lung destruction [35]. In this study, patients with a history of pulmonary TB were more than six times as likely to develop bronchiectasis, which is consistent with other studies [6, 20, 22].

A systematic review and meta‐analysis of 13 studies published between December 2019 and February 2020 has all shown that COVID‐19 is an emerging risk factor for bronchiectasis [27]. Our finding is also consistent with these studies, as it identified a significant association of COVID‐19 with bronchiectasis. Fibrosis and post‐COVID traction bronchiectasis contribute to mechanisms by which COVID‐19 causes bronchiectasis [36].

Cohort studies done in India [9] and Taiwan [20], have shown that asthma and COPD are risk factors for bronchiectasis. Our research has shown that both bronchial asthma and COPD are associated with bronchiectasis and is consistent with most of the studies.

Bronchial asthma is a state of chronic inflammation. This chronic inflammation may, in the long term, involve and damage the bronchi and cause irreversible damage [37]. Air trapping and mucus plugging also contribute. In patients with COPD, there is unopposed neutrophil elastase activity, which will cause progressive airway damage [38]. There is also chronic air trapping and mucus plugs, which will cause airway dilatation and progressive damage. Another possible mechanism is repeated chest infections and colonization by Pseudomonas aeruginosa [39].

The primary novelty and justification for our study lie in its specific focus on the Ethiopian context. Epidemiological patterns of disease can vary significantly due to genetic, environmental, and healthcare access factors. Unlike other similar studies, smoking status, age, and sex were not associated with bronchiectasis in this study, likely due to a low proportion of smokers, limited sample size, or potential sampling bias. Our research was conducted precisely to address an identified gap in local data, aiming to determine the most common etiologies and the strength of their association with bronchiectasis within our population. Therefore, although the risk factors may be known globally, their relative prevalence and impact in Ethiopia were not previously quantified, which is the key contribution of this work.

The implications of this research are twofold. From a public health perspective, it delivers an evidence‐based mandate to strengthen TB control programs, targeting the primary prevention of bronchiectasis at its most common source. For clinical practice, it serves as an alert to consider bronchiectasis early in patients with a history of TB or uncontrolled obstructive lung disease, facilitating timely diagnosis via CT and intervention to reduce permanent lung damage. Concurrently, it reinforces the essential role of guideline‐adherent management of obstructive airway diseases in mitigating the risk of these debilitating sequelae.

Our research has some limitations. The inability to incorporate additional variables that might be connected to bronchiectasis is one of the study′s weaknesses. During the pandemic, most patients with COVID‐19 had their first‐ever HRCT. Therefore, it is difficult to determine whether they developed bronchiectasis after COVID‐19 or they already had bronchiectasis and acquired COVID‐19 later. Other risks such as repeated childhood infections were not assessed as many adult patients may not accurately recall or have been diagnosed with specific childhood respiratory infections. Furthermore, establishing a definitive cause‐and‐effect relationship based on historical recall alone, without objective evidence from childhood, is methodologically difficult and could introduce recall bias. Regarding cystic fibrosis (CF), although it is a classic cause of bronchiectasis in other populations, it is exceptionally rare in Ethiopia and was not a leading consideration in our cohort.

## 5. Conclusion

Our study has found that pulmonary tuberculosis, COVID‐19 infection, COPD, and bronchial asthma are generally linked to the development of bronchiectasis, whereas sex, smoking, and alcohol use are not significantly associated with bronchiectasis.

## Author Contributions

B.S. and M.A.T.: conceptualization, writing original draft, and data curation. W.A.A. and B.S.: investigation, data curation, and data analysis. M.A.T. and B.S.: data interpretation, data curation, review and editing.

## Funding

No funding was received for this manuscript.

## Ethics Statement

The study was approved by St. Paul′s Hospital Millennium Medical College institution review board on 31/03/2022 with Approval Number IR23/300. The patients′ data are anonymous, and written informed consent was obtained from each participant. This study complied with the Declaration of Helsinki.

## Conflicts of Interest

The authors declare no conflicts of interest.

## Data Availability

The data that support the findings of this study are available from the corresponding author upon reasonable request.
